# Changes in Inflammatory Cytokines, Vascular Markers, Cell Cycle Regulators, and Gonadotropin Receptors in Granulosa Cells of COVID-19 Infected Women

**DOI:** 10.31661/gmj.v13i.3625

**Published:** 2024-10-07

**Authors:** Sina Vakili, Amirabbas Rostami, Bahia Namavar Jahromi, Morteza Jafarinia

**Affiliations:** ^1^ Infertility Research Center, Shiraz University of Medical Sciences, Shiraz, Iran; ^2^ Department of Internal Medicine, Faculty of General Medicine, Yerevan State Medical University after Mkhitar Heratsi, Yerevan, Armenia; ^3^ Shiraz Neuroscience Research Center, Shiraz University of Medical Sciences, Shiraz, Iran

**Keywords:** SARS-CoV-2, COVID-19, Inflammatory Gene, Cell Cycle

## Abstract

Background: COVID-19 infection can negatively affect multiple organ systems, including the reproductive system. Previous research has indicated altered levels of inflammatory markers in the reproductive tissues of women with chronic diseases. This study aimed to assess the expression of inflammatory, vascular, cell cycle, and gonadotropin receptor genes in the granulosa cells and oocytes of women with recent COVID-19 infection undergoing Assisted Reproductive Technology (ART), compared to healthy controls.Materials and Methods: The study involved 15 women who had tested positive for COVID-19 within three months of ART treatment and 15 age-matched healthy women as controls. Granulosa cells were collected during oocyte retrieval, and RNA was isolated to analyze gene expression using quantitative real-time PCR. The evaluated genes included inflammatory cytokines (IL-1B, TNF-α, IL-6, IL-8), vascular genes (VEGF, ANGPT1), cell cycle regulators (FOXL2, Cyclin D1, Cyclin D2, KLF4), and gonadotropin receptors (LHCGR, FSHR).Results: Results showed significantly higher expression of inflammatory cytokines in the granulosa cells of COVID-19 positive women, including IL-1B (4.2-fold), TNF-α (3.8-fold), IL-8 (2.5-fold), and IL-6 (3.2-fold). Vascular genes VEGF and ANGPT1 were also overexpressed, while FOXL2 was downregulated and Cyclin D1/D2 were upregulated in the study group. However, LH and FSH receptor expression remained similar between both groups.Conclusion: The present study demonstrates altered gene expression of inflammatory cytokines, vascular factors and cell cycle regulators in granulosa cells and oocytes of COVID-19 positive women undergoing ART. The dysregulated molecular pathways could potentially impair folliculogenesis and oocyte development in SARS-CoV-2 infected individuals.

## Introduction

The COVID-19 pandemic, instigated by the SARS-CoV-2 virus, has become one of the most significant public health crises in contemporary history. Although the respiratory implications of the virus are well-documented, emerging research indicates that COVID-19 may also have repercussions for reproductive health [1][2][3][4]. The virus gains entry into host cells through the angiotensin-converting enzyme 2 (ACE2) receptor, which is present in several non-respiratory tissues, including the testes, ovaries, and placenta [5][6]. This presence raises alarms regarding the potential negative effects of COVID-19 on fertility and pregnancy outcomes. Numerous studies have indicated the presence of SARS-CoV-2 in various female reproductive tissues, including the ovaries, placental tissues, amniotic fluid, and breast milk of infected individuals [7][8][9]. This indicates that the virus may spread to the female reproductive system. Additionally, studies reveal elevated inflammatory cytokines in the serum and follicular fluid of women with COVID-19 who are undergoing in-vitro fertilization (IVF) [10][11][12]. Excessive inflammation caused by COVID-19 infection may hinder oocyte quality and fertility potential, as successful reproduction relies on a healthy ovulatory process and a well-balanced follicular microenvironment [13][14].

The female ovary is a complex organ that contains follicles at various stages of maturation. Each menstrual cycle involves the recruitment and selection of dominant follicles, culminating in the ovulation of a mature oocyte. This intricately regulated process relies on specific interactions among ovarian follicular cells, neuroendocrine signals, paracrine factors, and the developing oocyte [15].

Granulosa cells that line the follicles and the oocyte they surround engage in bidirectional communication to synchronize essential processes such as cell proliferation, differentiation, and apoptosis necessary for folliculogenesis. Disruptions in the molecular interactions between granulosa cells and oocytes can adversely affect the maturation and quality of the oocyte [16]. Previous studies have reported changes in the expression of inflammatory cytokines, growth factors, metabolic enzymes, and cell cycle regulators in the granulosa cells and oocytes of women with specific chronic conditions and obesity. These molecular changes were associated with impaired oocyte competence and reduced fertility outcomes [17]. Since COVID-19 causes a pathogenic infection with significant inflammatory response [18], it is likely that the disease alters the ovarian transcriptome too. Identifying the genes that are abnormally expressed in granulosa cells and oocytes could shed light on the mechanisms behind reproductive issues caused by COVID-19. This study aimed to thoroughly assess key genes involved in inflammatory, vascular, sex hormones, and cell cycle pathways in granulosa cells from women with COVID-19 undergoing assisted reproductive technology (ART), compared to healthy controls.

## Materials and Methods

**Figure-1:**
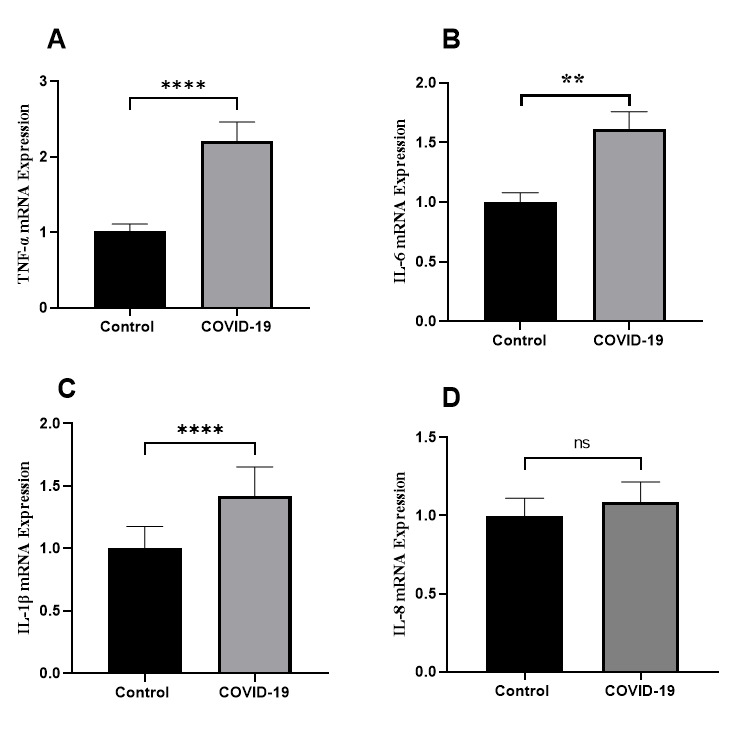
Figure 1. The mRNA levels of inflammatory cytokines TNF-α (A), IL-1β (B), IL-6 (C), and IL-8 (D). The results of the RT-PCR analysis unveiled a significant upregulation of inflammatory mediators including TNF-α, IL-1β, and IL-6 in the granulosa cells of COVID-19 positive patients. The results are expressed as the mean±standard deviation (SD). Statistical significance is indicated as **** for P<0.0001, *** for P<0.001, ** for P<0.01, and * for P<0.05. TNF-α: tumor necrosis factor-alpha, IL: interleukin.

Study Participants

This study involved 30 women receiving assisted reproductive technology (ART) treatment split into two groups: a study group of 15 women who tested positive for COVID-19 after ovarian stimulation, and a control group of 15 healthy women matched by age. Before beginning ovarian stimulation protocols, all ART candidates should not have a SARS-CoV-2 positive test.

This SARS-CoV-2 infection should also be checked on the day of the final oocyte maturation triggering prior to oocyte retrieval. Some patients have a positive SARS-CoV-2 test when ovary is stimulated and in the day of oocyte retrieval. Treatment is discontinued for these patients due to potential risks for embryo. Oocyte retrieval is advised, although, in a small number of individuals who are susceptible to developing severe ovarian hyperstimulation syndrome (OHSS) and its associated consequences, as well as in certain unique situations like endometriosis or breast cancer patients. Based on the current recommendations in infertility treatment centers, the collected follicular fluid and the cells from the OHSS patients are discarded [9]. For this investigation, unused samples from women infected with COVID-19 were gathered. Prior to the trial, patients were informed verbally and in writing, given enough time to think over their involvement, and provided their written consent. The Shiraz University of Medical Sciences Scientific and Ethics Committee gave its clearance to this study (approval code: IR.SUMS.REC.1400.672).

Stimulation and Granulosa Cells Collection

For every patient, a conventional GnRH antagonist protocol was followed. Following a transvaginal ultrasound (TVU) scan on the second day of the menstrual cycle, ovarian stimulation was initiated with HMG (PDHoMoG®, Pooyesh Daroo, Tehran, Iran) and Follitropin Alpha (Cinal-F®, CinnaGene, Alborz, Iran). On cycle day six, or when the leading follicle reached 12 mm, the GnRH antagonist (Cetrotide®, Injection, Powder, 250 μg, Serpero pharmaceutical, Italy) was started at a dose of 250 μg per day.

The drugs were kept up until the diameter of at least two follicles measured 17-18 mm. Subcutaneous injection of 2-3 ampules of Varian Pharmed’s Variopeptyl®, Injection, 0.1 mg, Tehran, Iran, a GnRH agonist, was then administered to initiate the last stage of oocyte maturation. Oocyte retrieval guided by TVU was carried out approximately 34-36 hours following triggering. Every patient’s excited follicle was recovered. Following a routine isolation process, the acquired follicular fluid was sent to the embryology lab in order to extract granulosa cells.

RNA Isolation and Gene Expression Analysis

Granulosa cells were processed immediately after collection to maintain RNA integrity. A commercially available RNA extraction kit was used for isolation. The quality and quantity of the extracted RNA were evaluated using spectrophotometry and gel electrophoresis. Only samples with high purity (A260/A280 ratio>1.8) and intact RNA profiles were chosen for further analysis. The isolated RNA was then converted into complementary DNA (cDNA) using a reverse transcription kit. The analysis concentrated on several key genes related to inflammation (IL-1B, TNF-α, IL-6, and IL-8), vascular function (VEGF and ANGPT1), cell cycle regulation (FOXL2, Cyclin D2, Cyclin D1, and KLF4), and gonadotropin receptor signaling (luteinizing hormone receptor (LHCGR) and follicle-stimulating hormone receptor (FSHR)).

After conducting qRT-PCR, the fold changes in gene expression between the study group (women with COVID-19 positive test) and the control group (healthy individuals) were determined using the 2-ΔΔCt method.

The data were normalized against the expression of the housekeeping gene GAPDH.

Statistical Analysis

Data were analyzed using SPSS (version 24, IBM, Chicago, IL) and GraphPad Prism (version 8). Differences in baseline characteristics between the study and control groups were evaluated with an independent samples t-test for continuous variables and a chi-square test (or Fisher’s exact test when appropriate) for categorical variables, with a p-value of less than 0.05 considered statistically significant.

Gene expression differences between the COVID-19 positive group and the control group were compared using the Mann-Whitney U test for non-normally distributed data or the Student’s t-test for normally distributed data.

The relationship between comorbidities and gene expression levels was examined using Pearson’s or Spearman’s correlation coefficients, depending on the distribution of the data.

## Results

**Figure-2:**
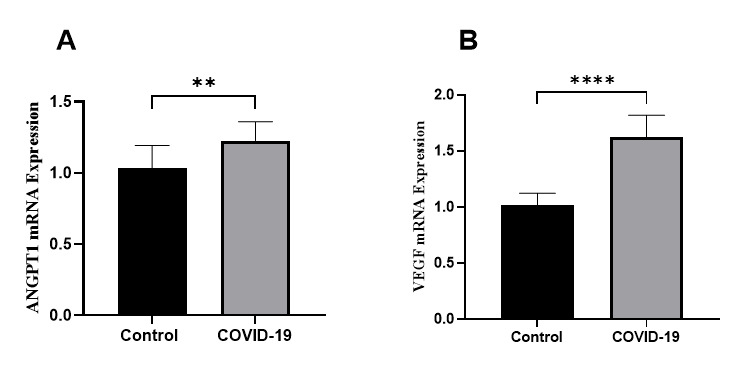
Figure 2. The mRNA levels of vascular markers ANGPT1 (A), and VEGF (B). The results of the RT-PCR analysis unveiled a significant upregulation of vascular markers including ANGPT1 and VEGF in the granulosa cells of COVID-19 positive patients. The results are expressed as the mean±standard deviation (SD). Statistical significance is indicated as **** for P<0.0001, *** for P<0.001, ** for P<0.01, and * for P< 0.05. ANGPT1: Angiopoietin 1, VEGF: Vascular endothelial growth factor.

1. Study Participants

The study group consisted of 15 women who had tested positive for COVID-19 after ovarian stimulation, with ages ranging from 26 to 38 years and a mean age of 31.2 years. The control group comprised 15 healthy women matched by age who were undergoing ART between 27 to 36 years old with the mean age of 32.1. There was no statistically difference between groups regarding the mean age.

2. Gene Expression in Granulosa Cells

2.1. Inflammatory Cytokines

The mRNA levels of inflammatory cytokines IL-1β, TNF-α, and IL-6 were significantly elevated in the granulosa cells of women with COVID-19 compared to the control group. Specifically, IL-1β expression was 1.4 times higher (P<0.0001), TNF-α was 2.2 times higher (P<0.0001), and IL-6 was 1.6 times higher (P<0.01). Although IL-8 expression also increased, this change did not achieve statistical significance (Figure-1)

2.2. Vascular Genes

Both the VEGF and ANGPT1 genes were found to be overexpressed in the granulosa cells of the study group. Specifically, VEGF mRNA levels were 1.6 times higher (P<0.0001), while ANGPT1 levels were 1.2 times higher (P=0.001) compared to the control group (Figure-2).

2.3. Cell Cycle

**Figure-3:**
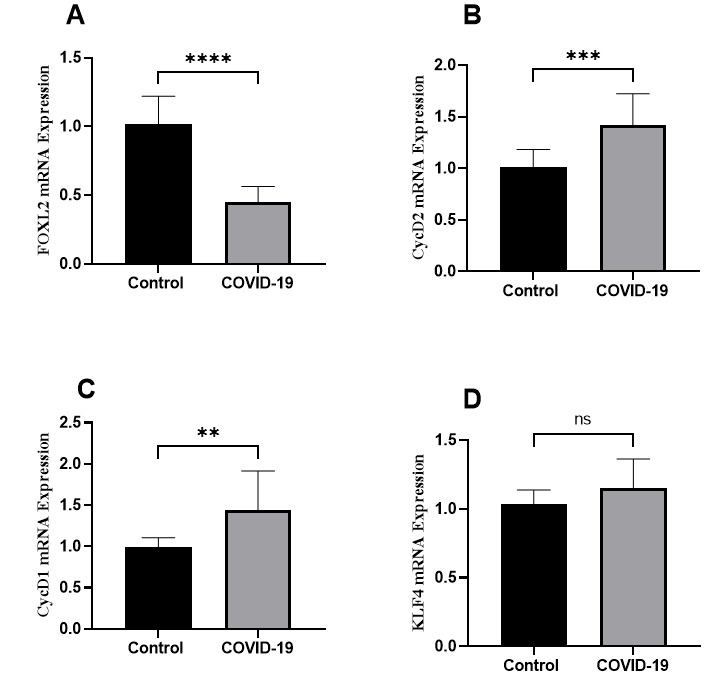
Figure 3. The mRNA levels of cell cycle markers FOXL2 (A), CycD2 (B), CycD1 (C), and KLF4 (D). The results of the RT-PCR analysis unveiled a significant upregulation of FOXL2 and a significant downregulation of cell cycle markers including CycD2, and CycD1 in the granulosa cells of COVID-19 positive patients. The results are expressed as the mean±standard deviation (SD). Statistical significance is indicated as **** for P<0.0001, *** for P<0.001, ** for P<0.01, and * for P< 0.05. Cyc: cyclin, KLF4: Krüppel-like factor 4.

The regulators FOXL2, a vital transcription factor for granulosa cell differentiation, exhibited a downregulation in women positive for COVID-19 (P<0.0001). In contrast, the cell cycle promoters Cyclin D2 and Cyclin D1 were upregulated by 1.4-fold (P=0.0001) and 1.4-fold (P=0.001), respectively. No significant change was observed in KLF4 expression (Figure-3).

2.4. Gonadotropin Receptor

**Figure-4:**
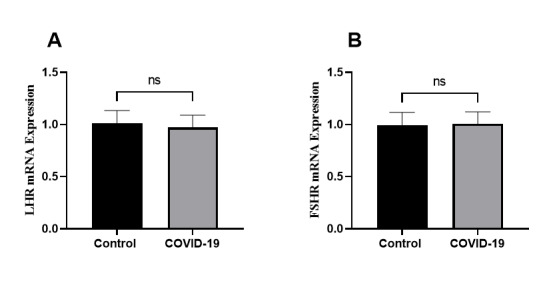
Figure 4. The mRNA levels of vascular markers LHR (A), and FSHR (B). The results are expressed as the mean±standard deviation (SD). Statistical significance is indicated as **** for P<0.0001, *** for P<0.001, ** for P<0.01, and * for P<0.05. LHR: LH receptor, FSHR: FSH receptor.

The expression of both LH (LHCGR) and FSH (FSHR) receptor genes in granulosa cells was similar between the study and control groups, showing no significant differences (Figure-4).

## Discussion

The findings of this study reveal significant alterations in the molecular profiles of granulosa cells obtained from women who tested positive for COVID-19 compared to healthy controls. Notably, there were significantly increased mRNA levels of inflammatory cytokines IL-1β, TNF-α, and IL-6 in the granulosa cells of women infected with SARS-CoV-2 undergoing ART. This aligns with earlier findings that COVID-19 infection triggers a strong inflammatory response marked by the release of cytokines [19]. Inflammatory cytokines such as IL-1β and TNF-α are known to disrupt ovarian function through various mechanisms, including inducing apoptosis in granulosa cells, interfering with steroidogenesis, and hindering oocyte maturation [20]. The increased levels of cytokines observed in this study suggest a pro-inflammatory ovarian microenvironment in women with COVID-19, which may negatively affect folliculogenesis and oocyte quality. Interestingly, although IL-8 exhibited a trend toward upregulation, it did not achieve statistical significance, indicating a differential regulation of cytokines in response to SARS-CoV-2 infection. Additionally, the vascular genes VEGF and ANGPT1 were found to be overexpressed in the granulosa cells of women who tested positive for COVID-19. VEGF plays a crucial role in promoting angiogenesis during follicle development by facilitating the formation of new blood vessels [21]. Increased levels of VEGF indicate heightened vascular permeability and angiogenesis, which may disrupt the blood-follicular barrier. The overexpression of ANGPT1, a crucial mediator of vascular stabilization, protection, and remodeling, indicates that significant vascular changes are occurring in response to SARS-CoV-2 infection. Dysregulated angiogenesis and vascular instability are known to negatively impact oocyte maturation [22][23]. Furthermore, the study found that FOXL2, a master regulator of granulosa cell differentiation, was downregulated, while cell cycle genes Cyclin D1 and D2 were upregulated in the granulosa cells of women infected with COVID-19. FOXL2 is essential for maintaining granulosa cell identity and coordinating folliculogenesis with proliferation signals [24][25]. Aberrant expression of FOXL2, coupled with excessive cell cycling, may result in failed differentiation and inadequate coordination of growth with developmental signals, ultimately hindering follicle development. This study is the first to report changes in key cell cycle mediators in relation to COVID-19 infection, offering new mechanistic insights. Notably, there were no differences in the gonadotropin receptor genes LH and FSH between the groups, suggesting that SARS-CoV-2 infection may not directly affect pituitary-ovarian communication. However, the disproportionate changes observed in other molecular regulators underscore the complex interactions among the inflammatory microenvironment, intra-ovarian signaling networks, and oocyte quality in the context of COVID-19.

A limitation of this study was its cross-sectional design, which prevents the assessment of long-term or recurring effects of COVID-19 on ovarian function and fertility potential over time. However, the current data provide new insights into how SARS-CoV-2 infection disrupts various ovarian pathways at the molecular level during the acute phase. By integrating clinical parameters of participants with gene expression patterns, a comprehensive profile analysis was achieved. Future prospective studies are needed to evaluate ovarian reserve markers and the number and quality of oocytes following infection.

## Conclusion

In conclusion, this research significantly enhances our understanding of the relationship between COVID-19 and female reproductive health. It reveals that SARS-CoV-2 dysregulates expression of inflammatory cytokines, growth factors, and cell cycle regulators persists in the granulosa cells, indicating long-term pathological changes. This molecular evidence supports the hypothesis that COVID-19 infection may disrupt follicular development and oocyte maturation through altered signaling within the ovary. The findings shed light on potential pathogenic mechanisms and have implications for counseling and managing fertility in women who have recovered from SARS-CoV-2. Further exploration of mitochondrial function, DNA repair capacity, meiotic resumption, and embryo development post-fertilization could provide deeper insights. Additionally, this study underscores the necessity to investigate therapeutic interventions, such as anti-inflammatory strategies, aimed at reversing COVID-19-related ovarian changes and optimizing fertility preservation in women of reproductive age who have been infected.

## Conflict of Interest

None.
